# Prediction of survival of diffuse large B‐cell lymphoma patients via the expression of three inflammatory genes

**DOI:** 10.1002/cam4.714

**Published:** 2016-07-09

**Authors:** Shuangtao Zhao, Nan Bai, Jianlin Cui, Rong Xiang, Na Li

**Affiliations:** ^1^ School of Medicine94 Weijin RoadTianjin300071China; ^2^ Collaborative Innovation Center for BiotherapyNankai University94 Weijin RoadTianjin300071China; ^3^ Prenatal Diagnosis CenterThe First Affiliated Hospital of Zhengzhou UniversityZhengzhouHenan450052China; ^4^ Tianjin Key Laboratory of Tumor Microenvironment and Neurovascular RegulationTianjin300071China

**Keywords:** CSF3, diffuse large B‐cell lymphoma, IL1A, IL6, tumor‐associated macrophages, tumor microenvironment

## Abstract

Currently, several gene‐expression signatures that were used to predict survival of diffuse large B‐cell lymphoma (DLBCL) patients, showed a restriction on the practical work for lack of convenient operation. In this study, we screened inflammatory genes whose expression correlated with survival of DLBCL and established a predictive model including *IL6, IL1A* and *CSF3* through multivariate Cox regression based on the expression of these three genes. We validated the model at protein level in our clinical serum cohort composed of 101 patients of DLBCL and 50 healthy controls and 534 DLBCL patients at mRNA level from three independent Gene Expression Omnibus (GEO) data sets. We found our model to be independent of the International Prognostic Index (IPI), moreover, it can augment the predictive power of IPI. In summary, our three‐gene model is sufficient to predict survival of DLBCL patients via measuring the concentration of three inflammatory cytokines in peripheral blood.

## Introduction

Diffuse large B‐cell lymphoma (DLBCL) is the most common subtype in non‐Hodgkin lymphoma (NHL) and a biologically heterogeneous group of tumors, which comprises 30–35% in all NHLs 1. More than 50% of DLBCL patients could have long‐term disease‐free survival with combination therapy although this tumor is biologically aggressive 2. However, approximately one‐third of the patients relapse or are refractory to the standard treatment, and eventually succumb to this disease 3. In clinical practice, the standard chemotherapy regimen of cyclophosphamide, doxorubicin, vincristine, and prednisone (CHOP), and the addition of rituximab to CHOP (R‐CHOP) have significantly improved the survival of patients 4, 5. The International Prognostic Index (IPI), as one of the most important factor to predict the outcome of DLBCL patients, includes five factors such as patient age, Ann Arbor tumor stage, serum concentration of lactate dehydrogenase, performance status, as well as a number of involved extranodal disease sites 6. However, some have questioned the validity of the IPI in the outcome of patients with DLBCL, as it does not fully represent disease heterogeneity 7. Therefore, due to increased diagnostic and therapeutic sophistication, efforts have transferred to discover novel biomarkers correlated with prognosis which stratify patients according to risk factors, thereby providing a basis for individually tailored treatment.

Lossos et al. studied 36 genes whose expressions were reported to predict prognosis of DLBCL 8. By using real‐time RT‐PCR to detect the mRNA expression level of each gene from independent samples of 66 patients and applying the univariate analysis, they selected out six genes including *LMO2, BCL6, CCND2, FN1, SCYA3*,* a*nd *BCL2* based on the rank of the gene's prediction ability and constituted a prediction model based on their mRNA expression level, which was independent of IPI and sufficient to predict the survival of DLBCL patients 8.

Recently, a significant correlation has been shown between tumor microenvironment (TME) and risk and prognosis in patients with various types of cancers 9, 10, 11, 12, 13. It becomes obvious that the microenvironment plays an important role in tumor progression. Some research efforts indicate that cytokines and immune‐related proteins play significant roles in predicting the risk of non‐Hodgkin lymphoma. Rosenwald et al. reported a prediction model composed of 17 genes for the survival in CHOP‐treated DLBCL patients, most of these genes were expression signature characters for germinal‐center B cells, proliferation, lymph node, or class II major histocompatibility complex molecules 14]. The tumor microenvironment contains many inflammatory cytokines such as IL6, IL1A, IL8, G‐CSF/CSF3, and CCL3, etc. It is also populated by notable tumor‐associated macrophages (TAMs), whose fundamental role is to mediate the chronic inflammatory response correlated with cancer. It is demonstrated that macrophages possess anti‐tumor or tumor‐promotion effects, depending on their acquired immune‐phenotype (M1 or M2) which express different levels of chemokines, cytokines. TAMs (M2 phenotype) predict poor outcome in DBLCL patients‐treated with chemotherapy 15, 16, 17. Polymorphisms of host cytokines and immunity‐related genes were reported to play very important roles in predicting survival of DLBCL patients 7. It was shown that these immune genes appeared to influence differentiation, proliferation, and metastasis of both tumor as well as stromal cells, regulated communication between tumor and stroma, and modulated interactions between tumor cells with the extracellular matrix 18.

Several clinical studies indicated that the cancer cachexia syndrome contributed to morbidity and mortality in more than 80% advanced patients 19, with proinflammatory cytokines being the most important factors for its genesis 20. Furthermore, it was reported that proinflammatory cytokines such as IL6 might be useful as prognostic markers 21. The elevated serum IL6 level could predict a poor survival in patients with DLBCL 21. Other cytokines, including IL10 haplotype, IL1A, and TNF were reported to be among the strongest predictors of overall survival in patients with DLBCL 7. Meanwhile, prophylactic application granulocyte‐colony stimulating factor (G‐CSF/CSF3) was observed to improve clinical outcome of elderly DLBCL patients receiving CHOP chemotherapy 22. Other cytokines, such as IL8, IL10, etc. associated with DLBCL 23. *CCL2* and *CXCL10*, etc. were also reported to be correlated with the survival of lymphoma patients 24.

In this study, five genes (*IL6, IL1A, IL8, CCL3*, and *CSF3*) were selected from overlaps between inflammatory genes and the survival‐related genes of DLBCL in U937, which underwent significant changes after co‐culturing with OCI‐LY3 cells. They were then subjected to test in 101 clinical patients with DLBCL and 50 healthy controls. Subsequently, a novel predictive model based on three genes (*IL6, IL1A,* and *CSF3*) was constructed in our clinical cohort, which was independent of the IPI, but could predict the overall survival of DLBCL patients. In addition, we validated the predictive model in three other independent patient cohorts from GEO data sets and found our predictive model was superior to the one‐gene model in the diagnostic value for prognosis. Thus, we constructed an effective and amenable simple assay model which could be applied in clinical practice routinely.

## Materials and Methods

### Cell lines and cell culture

U937 (a human monocyte‐macrophage cell line) was obtained from the Cell Resources Center of the Biological Sciences Institute in Shanghai of the Chinese Academy of Sciences in 2011. They were maintained into culture media composed of RPMI‐1640, 10% fetal bovine serum (FBS), 100 *μ*/mL penicillin, and 0.1 mg/mL streptomycin. For the co‐culture system, 3 × 10^5^ human DLBCL cell line‐OCI‐LY3 cells were plated in 6‐well plates, 1 × 10^5^ U937 cells were seeded in each Boyden Transwell chamber (0.4 *μ*m pores, Millipore, Billerica, MA). The U973 cells without the OCI‐LY3 co‐culture was used as control. Four days later, the U937 cells of the two groups were collected, respectively, and subjected to RNA sequence.

### RNA‐Seq

RNA was isolated from U937 cells using Trizol LS (Invitrogen, Carlsbad, CA) according to the manufacture's description. Subsequently, 100 *μ*g total RNA samples from these two groups were treated according to the Illumina protocols, respectively. During the quality control (QC) step, the sample library was qualified and quantified by Agilent 2100 Bioanaylzer and ABI Step One plus Real‐Time PCR System. All genes were analyzed by screening differentially expressed genes (DEGs) among samples. “False Discovery Rates (FDR) ≤ 0.001 and | log_2_^Ratio^ (Ratio = U937_co‐culture_/U937_untreated_) | ≥1” were used as the thresholds to judge the significance of gene expression differences.

### Healthy controls and patients

This research was completed according to the approved guidelines of the Institute Research Ethics Committee of the Chinese People's Liberation Army General Hospital. All 101 patients and 50 healthy controls from this hospital between 2013 and 2014 provided informed written consent for the study of these samples and their use in research. All samples were analyzed in a blinded manner. Serum samples of healthy controls were collected before any treatment, without fever within 7 days, without known pregnancy, and without a history of any chronic or acute illnesses. Diagnosis of DLBCL was double confirmed by the WHO 2008 lymphoma classification 25 for all patients before their participation in this study. Serum samples from patients were collected after chemotherapy (CHOP or Rituximab‐ CHOP regimens) or before chemotherapy. The tumor stages of the lymphoma were recorded according to Ann Arbor staging.

### Immunohistochemistry

Slides of formalin‐fixed, paraffin‐embedded tissues from our clinical cohorts were stained with anti‐body CD163 (1:50; Leica Biosystems, Wetzlar, Germany), Immunohistochemistry was performed with an automated staining system (Dako Autostainer, Dako, Carpinteria, CA).

All immunostained slides were submitted to virtual microscope scanning under the high‐power magnification objective (40×), using a ScanScope CS2 eSlide (Aperio Technologies, Vista, CA). For enumeration of immune cells, four different fields were captured from virtual microscopic images. The level of TAMs infiltration was quantified as the ratio of CD163^**+**^ cells versus total cells in each image field. The mean value for each staining was averaged from that of four image fields.

### Serum collection and detection

From each patient, a total of 10 mL of peripheral blood sample was collected into tubes containing a separating gel and clot activator, and then transferred into new tubes after centrifuging at 1939 g for 7 min, followed by storage in aliquots at −80°C to avoid repeated freeze thawing before cytokines detection.

Serum cytokine concentrations were measured by using a multiplex bead‐based sandwich immunoassay kit (Millipore, Billerica, MA) that contained fluorescence‐coated magnetic beads. These cytokines were detected according to the instructions of the manufacture. Fluorescent signals of the beads were recorded with a Luminex 200 system. The interassay coefficient of variability (CV) and intra‐assay CV ranged from 3.7 to 17.0% and 4.6 to 11.0%, respectively.

### Statistical analysis

The relative expression values of the 29 survival‐related genes were normalized via univariate analysis, and five genes (*IL6, IL1A, IL8, CCL3*, and *CSF3*) were incorporated into the study with an univariate Z score> 1.2 or <−1.2. Expression of these five genes in serum of 101 DLBCL patients were log‐transformed (on a base 2 scale) in a manner similar to the transformation of RNA array data later and compared with 50 healthy controls by the Wilcoxon rank test.

In order to construct a model which predicts survival, we performed survival analyses using the Kaplan–Meier method, log‐rank tests and univariate proportional hazards analysis before multivariate Cox regression. Subsequently, we analyzed the five genes in a multivariate Cox proportional‐hazards regression model, with overall survival as the dependent variable. Then, three genes (*IL6, IL1A,* and *CSF3*) were selected into the final model, whose values were multiplied by a risk parameter derived from the multivariate analysis. The survival time was calculated from the date of diagnosis until death or the last follow‐up contact. The survival curves were estimated by the Kaplan–Meier product‐limit method and compared by the log‐rank test and trend test. All the factors of patients were included in the model such as time, status, age, gender, Ann Arbor, chemotherapy status, values of five genes, and so on. Two‐sided *P* values had to be <0.05 to indicate statistical significance.

To validate the availability of this model, we applied it into three independent GEO data sets previously published with gene‐expression data for DLBCL. Every gene‐expression value in each data set was statistical significant (*P* < 0.05). To compare the diagnostic values for prognosis among our predictive model, IPI, *IL6, IL1A*, and *CSF3*, we established receiver operating characteristic (ROC) curves to evaluate them, and compared the areas under the ROC by Z‐scores. We also used Youden index to choose the cutoff value that determined the sensitivity and specificity.

## Results

### Seven common genes were found between survival and inflammatory genes database

**Figure 1:**
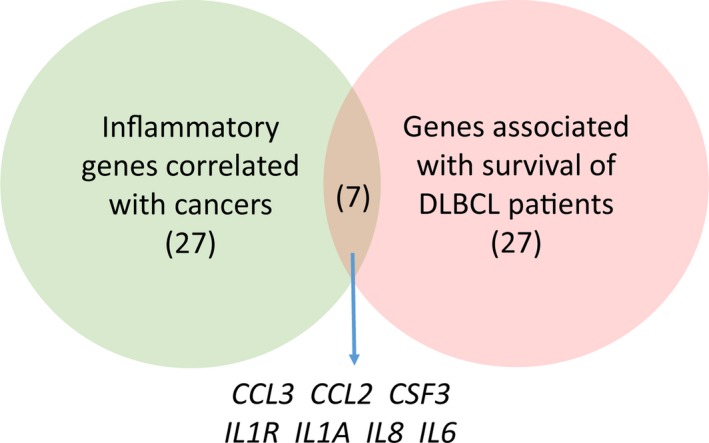
Overlaps between survival‐related genes of DLBCL and cancer‐associated inflammatory genes. The Venn diagram showed the overlap between genes which predict survival of patients with DLBCL and the inflammatory genes correlated with cancers. The number of genes in each category and the seven overlapping genes were indicated.

To determine the correlation between inflammatory genes and genes correlated with the survival of DLBCL, 34 survival genes from the previous study which were measured with microarrays or quantitative RT‐PCR either individually or in large data sets to predict survival in DLBCL patients were found 8. 34 inflammatory cytokines from the opened results of research were included in this study, which could mediate direct interactions between cells and regulate processes taking place in the extracellular environment, and play a significant role in the multi‐faceted response to inflammatory disease as well as cancers 7, 22, 23, 26, 27, 28, 29. We found seven common genes from these two databases, including *IL6, IL1A, IL8, CCL3, CCL2, IL1RN*, and *CSF3*, have the predictive power for the prognosis of DLBCL patients (Fig. 1).

## 29 survival‐related genes were selected for testing

**Figure 2:**
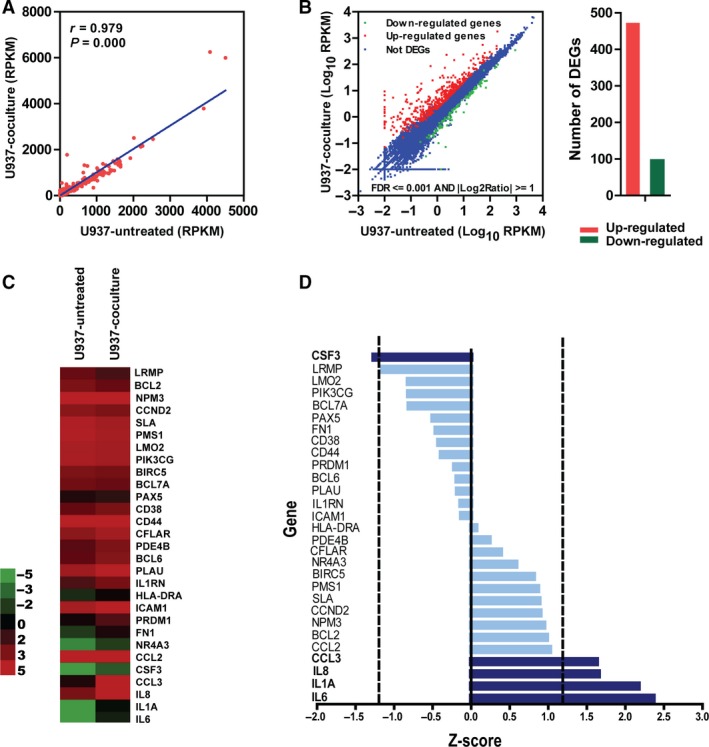
Univariate analysis of 29 survival genes with relative expression as a dependent variable. (A) Correlation of mRNA expression changes between the U937‐untreated group and the U937‐coculture group. RPKM, reads per kilobase per million mapped reads. Correlation coefficient *r* = 0.979, *P *=* *0.000 (Spearman's correlation). (B) Gene expression level of U937‐untreated and U937 co‐culture group. The left panel: Red dots represented up‐regulated genes, the green ones represented down‐regulated genes, and the blue ones represented genes that were not differently expressed. The right panel: quantification of up‐regulated genes and down‐regulated genes in the RNA sequence. The numbers are 470 and 97 separately. (C) Heat map depicting RNA expression profiling of 29 survival genes with DLBCL between the U937‐untreated and the U937‐coculture group. (D). These genes were ranked according to their predictive power (univariate Z score), a negative or positive score correlates with longer or shorter overall survival. The dashed lines indicated an univariate Z score of ±1.2. Five genes were included in the multivariate Cox regression to screen the predictive genes.

Recent studies demonstrated the important function of TAMs in the progression of cancers 15, 16, 17, 30. Hans et al. divided DLBCL into the germinal center B‐cell like (GCB) and activated B‐cell like (ABC) subtypes on the basis of immunohistochemical findings. Generally, the ABC type shows worse prognosis as compared with the GCB type 31. In order to determine the cytokines and chemokines involved in the interaction between DLBCL and TAMs, that impact prognosis of DLBCL patients, we cultured U937 cells with or without OCI‐LY3, cells which belongs to the ABC type, for 4 days. Then, the mRNA from each group was collected and subjected to RNA sequence. From the result, we deduced that RNA expression of these two groups significantly correlated with each other (*r* = 0.979, *P *=* *0.000; Fig. 2A). The expression of 567 genes had been changed, which included 470 up‐regulated genes and 97 down‐regulated genes (Fig. 2B). Among the 567 genes we discovered, the expression of 29 survival‐related including 7 inflammatory genes showed a significant difference (*P *<* *0.05), and five of these survival‐related inflammatory genes (*IL6, IL1A, IL8, CCL3*, and *CSF3)* were more up‐regulated than the other two genes after co‐culture with OCI‐LY3 cells (Fig. 2C). The relative‐expression values of 29 survival genes were normalized via univariate analysis as a dependent variable (Fig. 2D). These genes were ranked on the basis of their predictive power (univariate Z score of Log_2_^Ratio^) (Fig. 2D). A positive Z score was associated with the relative expression value of gene which indicate poor prognosis, and a negative Z score was associated with the relative value of the gene which correlated with good prognosis. By inspection, an optimal number of genes were selected to construct a predictive model from this ranking. We observed only two gene inclusions in our study according to the conventional cutoff value for Z of ±2.0 (*P *<* *0.05). Thus, we chose the Z value of ±1.2 (*P *=* *0.23), which allowed five genes to be inclusive, as they exceeded the absolute Z value of 1.2 in the univariate analysis, including *IL6, IL1A, IL8, CCL3*, and *CSF3* (Fig. 2D).

**Figure 3:**
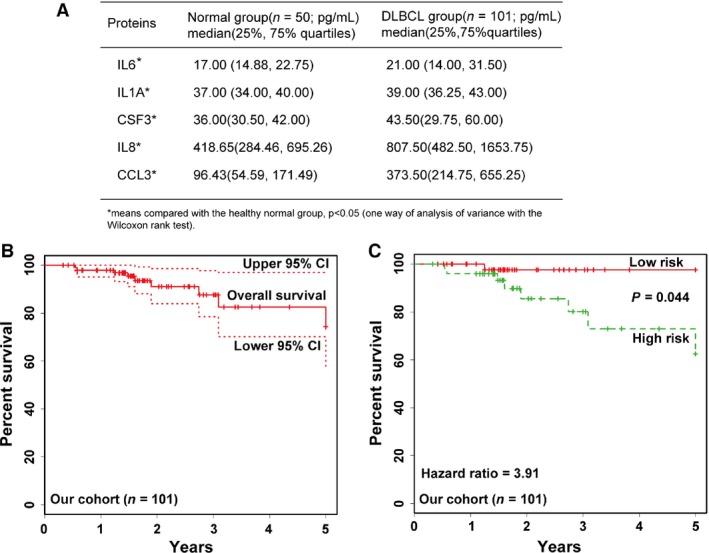
Validation of the performance of three‐gene model in our clinical cohort. (A) Comparison of the concentration of cytokines in healthy controls and all DLBCL patients. *P *<* *0.05 (Wilcoxon rank test). (B**)** Kaplan–Meier estimates of overall survival in the 101 patients with DLBCL. The dotted lines represent 95 percent confidence intervals. (C). Kaplan–Meier curves for overall survival in the low‐risk and high‐risk group as defined by our predictive model based on the relative expression of three genes (*IL6, IL1A* and *CSF3*). The hazard ratio between the two groups is 3.91. *P *=* *0.044 (Log‐rank test).

Previous studies demonstrated the abundance of translating mRNAs correlated positively with that of proteins, but negatively with the length of mRNAs in tumor cells 32. Since the mRNAs for these five genes were relatively short, we then determined the possibility of using these five proteins to develop a novel predictive survival model. The expressions of these five genes were subsequently detected in 151 serum samples from 50 healthy controls and 101 patients by multiplex microbead immunoassay. These two groups in serum protein data were compared by one‐way of analysis of variance with the Wilcoxon rank test, which showed significantly up‐regulated expression for all these five genes in patients than the normal group (*P *<* *0.05, Fig. 3A**)**. This indicated that the patients included into our study were different from the normal controls, and that all the serum samples from patients with lymphoma were feasible for constructing a predictive model of DLBCL patients with these genes.

### A three‐gene model was constructed for predicting survival of DLBCL patients

When the overall survival as a dependent variable was applied into a multivariate Cox regression analysis, only the *IL6* gene predicted the overall survival independently (*P *<* *0.05, Table S1). The forward stepwise method of multivariate Cox regression analysis was then performed, which included time, status, age, gender, Ann Arbor, genotype, and the expression of five genes except for IPI (since IPI overlaped with age and Ann Arbor). The expression of *IL6, CSF3*, and *IL1A* showed significant difference (*P* values of 0.007, 0.040 and 0.070 respectively, Table S1). As a result, a model independent of IPI was developed based on a risk parameter of each of the three genes in the Cox regression analysis. This could be described by the following equation: Mortality‐prediction score (*Y*) = (1.988 × *IL6)* + (0.683 × *IL1A*) + (−0.762 × *CSF3*). For example, the negative risk parameter of *CSF3* indicates that its higher expression is correlative with longer survival. In contrast, the positive parameter of *IL6* indicates its higher expression that correlates with shorter survival.

For failure‐free and overall survival, these 101 patients were stratified by their mortality‐prediction scores and were divided into two groups according to whether they had a low and high risk of mortality (lower risk ≤ 8.172; high risk > 8.172). According to the risk groups, we showed the clinical characteristics of the patients in Table**** 1. The rates of overall survival at 5 years were 74.27% for all patients (95% confidence interval‐CI: 57.10–96.50%, Fig. 3B), and showed 97.62% (95% CI: 93.12–100%) in the low‐risk group, and 62.47% (95% CI: 41.70–93.60%) in high‐risk group, respectively (Fig. 3C, *P* = 0.044). The hazard ratio (HR) between these two groups was 3.91 (Fig. 3C), which means that patients in the high‐risk group were at 3.91 times the risk of those in the low‐risk group. These results supported the efficiency of this novel predication model.

**Table 1:** Clinical characteristics of the DLBCL patients^a^

Characteristic	Low risk group (*n* = 51)	High risk group (*n* = 50)	Total (*n* = 101)
Age (years)			
Mean	47.88	55.96	51.88
Range	16–78	15–85	15–85
Sex, *n* (%)			
Male	27 (52.9)	32 (64.0)	59 (58.4)
Female	24 (47.1)	18 (36.0)	42 (41.6)
Ann Arbor, *n* (%)			
I	5 (9.8)	4 (8.0)	9 (8.9)
II	9 (17.6)	7 (14.0)	16 (15.8)
III	13 (25.5)	21 (42.0)	34 (33.7)
IV	24 (47.1)	18 (36.0)	42 (41.6)
Types, *n* (%)			
GCB	24 (47.1)	27 (54.0)	51 (50.5)
Non‐GCB	27 (52.9)	23 (46.0)	50 (49.5)
Chemotherapy, *n* (%)			
Yes	49 (96.1)	45 (90.0)	94 (93.1)
No	2 (3.9)	5 (10.0)	7 (6.9)
IPI, *n* (%)			
0~2	29 (56.9)	27 (54.0)	56 (55.4)
3~5	22 (43.1)	23 (46.0)	45 (44.6)

a Patients in the two groups have mortality‐predictor score (*Y*) as follows, low‐risk group: *Y* ≤ 8.172, and high‐risk group: *Y* > 8.172.

GCB, germinal center B‐cell like; IPI, International Prognostic Index

### The three‐gene model was in line with CD163^+^ TAMs infiltration

**Figure 4:**
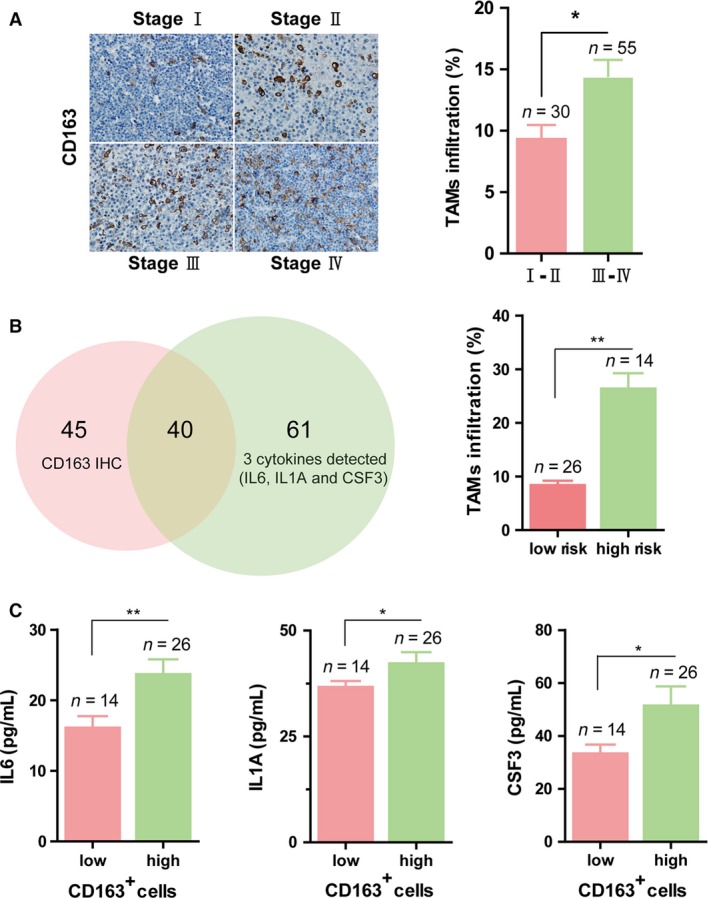
The three‐gene model was in line with CD163^+^ TAMs infiltration. (A) Left panel: representative images of tumor‐infiltrating CD163^+^ cells in different Ann Arbor stages. The right panel: comparison of the percentage of CD163^+^ cells (TAMs) in Ann Arbor stages at stage I and II and those at III and IV (*t*‐test, data presented as mean + SEM). (B) Left panel: a Venn diagram revealed the overlap between patients with Immunohistochemistry staining of CD163 and patients with the three cytokines detected. Right panel: the bar plot showed the TAMs infiltration were significantly more in the high‐risk group than in low‐risk group (*t*‐test, data presented as mean + SEM). (C) The concentrations of three cytokines in serum were positively correlated with the CD163^+^ cells (represent for TAMs infiltration). The bar plots showed that the value of each cytokine concentration was significantly higher in the group with high amount of CD163^+^ cells (cutoff value = 9%) than in the low one (*t*‐test, data presented as mean + SEM). *indicates significant difference with *P* < 0.05, **indicates significant difference with *P* < 0.01.

It was reported that an increased number of CD163^**+**^ macrophages had a lymphoma‐promoting function in DLBCL and predict poor clinical outcome 17. To determine whether CD163^**+**^ macrophages profiles were able to define low‐ and high‐risk populations in our clinical cohort, we evaluated the CD163 protein expression by using immunohistochemistry assay on 85 samples corresponding to DLBCL. From the results, we found the percentage of CD163^**+**^ cells was significantly higher in Ann Arbor stage III and IV than stage I and II (Fig. 4A), which was also in line with the risk level of the three‐gene model (Fig.4B). That means the outcome of patients with DLBCL would be worse in high levels of TAMs infiltration, and the similar result was that the probability of mortality or relapse of patients with DLBCL would be higher and higher following the predictive scores increasing. In addition, the serum concentration of IL6, IL1A, and CSF3 increased in DLBCL patients with high CD163^**+**^ cells percentage (Fig. 4C). So we surely regarded the three‐gene model as an effective way to predict prognosis in DLBCL patients.

### The three‐gene model was validated in GEO data sets

To further validate this predictive survival model at the mRNA level, 411 microarray samples from GSE10846 were analyzed. As shown in Figure S1, in the entire GSE10846 series patients, we found that DLBCL patients with high risk scores tended to express high levels of risky genes (*IL6* and *IL1A*) in their tumors, whereas patients with low risk scores tended to express high levels of protective genes (*CSF3*). It means the model derived from serum samples could also be applied in the microarray samples.

**Figure 5:**
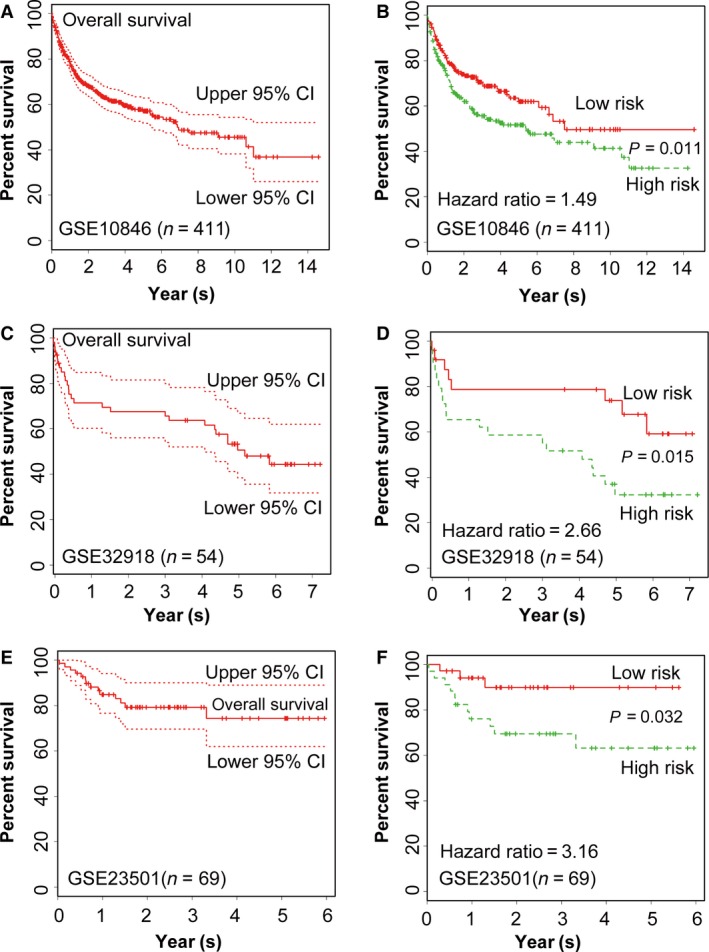
Validation of the performance of three‐gene model in GSE10846, GSE32918, and GSE23501. Kaplan–Meier estimates of overall survival in the 411 patients with DLBCL reported in GSE10846 (A), the dotted lines represent 95 percent confidence intervals. Similar estimates of the data on GSE32918 (C), and GSE23501 (E) were also shown. Kaplan–Meier estimates of overall survival in DLBCL patients reported in GSE10846 (B), GSE32918 (D) and GSE23501 (F), the hazard ratio between low risk and high risk group is 1.49, 2.66 and 3.16, respectively in each cohort. *P = *0.011, 0.015 and 0.032 separately (Log‐rank test).

In order to detect the validity of the model, we applied it to previously published microarray gene‐expression data from GSE10846 12, GSE32918 33 and GSE23501 34. The significance analysis of microarrays was also applied into the GEO data sets in order to identify these inclusive three genes that were useful for this new model. This was a supervised method for identification of genes with possible statistically significant association with patient survival. As a result, 411, 54, and 69 patients of DLBCL were selected from GSE10846, GSE32918 and GSE23501, respectively. As for all the patients, the rates of overall survival at 5 years were 57.0% (95% CI: 51.70–62.90%) in GSE10846 (Fig. 5A), and were 61.90% (95% CI: 54.1–71.0%), and 50.30% (95% CI: 43.10–58.70%), respectively (*P *=* *0.011, Fig. 5B) in the low‐ and high‐risk group. The median survival times were 7.49 years (95% CI: 6.64–NA) and 5.40 years (95% CI: 2.59–11.00) in the low‐ and high‐risk group, respectively. The hazard ratio (HR) between the two groups was 1.49. Similarly, the rates of overall survival at 5 years were 50.60% (95% CI: 38.40–66.80%) for all patients in GSE32918 (Fig. 5C) and 74.30% (95% CI: 62.00–89.00%) for patients in GSE23501 (Fig. 5E); the rates of the overall survival at 5 years were 67.60% (95% CI: 50.10–91.20%) and 32.3% (95% CI: 18.70–56.00%) in the low‐ and high‐risk group, respectively, in GSE32918 (*P *=* *0.015, Fig. 5D), and 89.80% (95% CI: 79.30–100%), and 63.01% (95% CI: 47.00–84.80%), respectively, in those of GSE23501 (*P *=* *0.032, Fig. 5F). The HR between the two groups was 2.66 in GSE32918 (Fig. 5D) and 3.16 in GSE23501 **(**Fig. 5F). The median survival times were >7.21 years and 4.08 years (95% CI: 1.28–4.34), respectively, for low‐ and high‐risk groups in GSE32918, and were >5.96 years for both groups in GSE23501. Therefore, these tests confirmed that as well as in our cohort, this three‐gene model was effective in predicting patients' prognosis in the three microarray data sets.

### The three‐gene model added prognostic value beyond that of the IPI, and showed superiority in the predictive effectiveness to the one‐gene model

**Figure 6:**
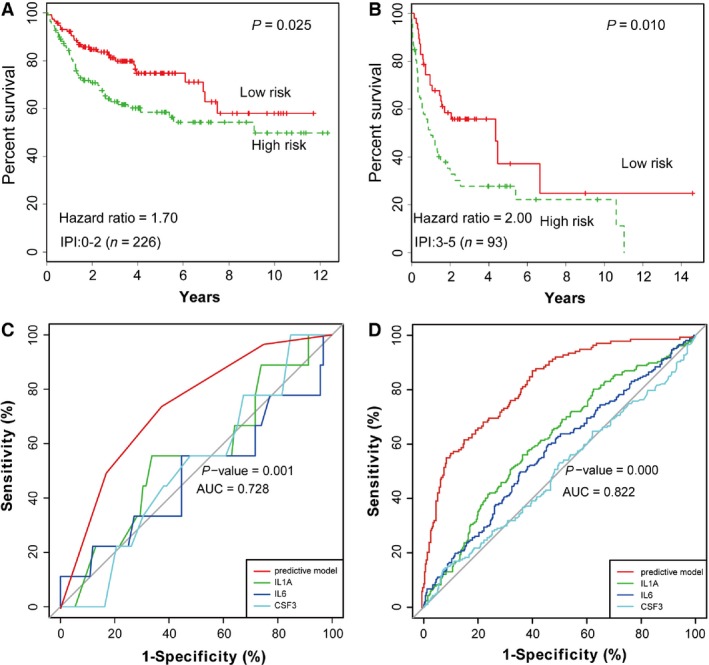
The three‐gene model added predictive power for IPI, and was superior to the one‐single‐gene model. The Kaplan–Meier estimates curves showed overall survival for groups of patients with low IPI risk scores (A) and high IPI risk scores (B), which were subdivided into two groups (low risk and high risk) according to our three‐gene predictive model. *P *=* *0.025, 0.010, respectively (Log‐rank test)**.** The AUCs of our predictive model were 0.728 (*P *=* *0.001) and 0.822 (*P *=* *0.000) in our cohort (C) and 411 patients in GSE10846 (D), respectively. Compared with the AUC of single gene‐*IL6, IL1A*, and *CSF3* model separately, the AUCs of our predictive models showed significant improvements (*P *<* *0.05,one way ANOVA test).

Subsequently, we investigated whether the three‐gene model could add prognostic value beyond that of the IPI. Among clinical patients in our sample, we divided them into two groups according to their IPI score (low IPI: 0–2; high IPI: 3–5), and further subdivided the patients in each group into two subgroups according to the prediction score from the three‐gene model. However, there were too few patients in the high risk of death subgroup in our samples for our result to achieve statistical significance. Therefore, we analyzed the larger data set published in GSE10846 to investigate the added value of the three‐gene model for IPI (Fig. 6A and B). Similarly, we first divided the samples of GSE10846 into two groups based on their IPI scores. Then, we subdivided each group into special levels of probability of survival with low‐ or high‐risk prediction scores from the three‐gene gene model (*P *<* *0.05, Fig. 6A and B). A group with an especially low probability of survival could be identified in each stratum of the IPI (Fig. 6A and B, green lines). Thus, by identifying the patients who had high‐risk scores from the three‐gene model in the high IPI score group (Fig. 6B), it was possible to identify the group of approximately 37.63% of patients with high IPI score who had especially short survival (Fig. 6B). Meanwhile, patients with low IPI group could be evaluated with specific proportion (19.47%) in the same way as above (Fig. 6A). Generally, we concluded that the three‐gene model could be used to predict survival of DLBCL patients independently and added the predictive power of the IPI. The independence between our predictive model and the IPI was further verified by multivariable Cox regression analysis in our cohort and GSE10846, in which our predictive model was a significant factor in both our cohort and GSE10846 data sets (*P *<* *0.05,). However, IPI, as an important prognostic factor, was not significant in our cohort (*P *=* *0.057, Table S2).

To identify the diagnostic value in individual prognosis, the ROC (receiver operating characteristic curve) method was used to compare the power among *IL6, IL1A*,* CSF3* and the three‐gene predictive model. Among our 101 patients, the area under the curve (AUC) of multivariate Cox regression was 0.728 (*P *=* *0.001) for our three‐gene model, which was higher than the value of 0.450 (*P *=* *0.625), 0.513 (*P *=* *0.900), 0.518 (*P *=* *0.858) for the one‐gene model of *IL6, IL1A*, and *CSF3*, respectively, in our clinical serum sample cohort (Fig. 6C). With a cutoff value of 3.5, the sensitivity and specificity of the predictive model were 80.00% and 78.70%, respectively. So, the result confirmed that the three‐gene model had superiority in the predictive effectiveness. We also detected the AUC of these different models in 411 patients from GSE10846 microarray data sets, and obtained similar results as above (Fig. 6D). Thus, this indicated that the three‐gene model which was approaching the predictive range needed for clinically useful tests, predicted overall survival better than the one‐gene model by comparing the predictive power (Fig. 6C and D). Collectively, these results demonstrated that our new predictive model was sufficient to work as a practical clinical risk tool for DLBCL patients.

## Discussion

As a subtype of non‐Hodgkin lymphoma (NHL), DLBCL is one of the most commonly diagnosed tumors in the Western countries. Although this type of lymphoma treatment is one of successful examples of modern therapy for tumors, the outcome for the patients is heterogeneous. Most relapses occur within 2–3 years after standard chemotherapy 21. Thus, it is significantly important to utilize prediction factors for the identification of patients with potentially bad prognosis, thus guiding the clinical treatment strategy.

Numerous biological models composed of prognostic markers have been proposed in patients with DLBCL, but many of them are not widely applied in virtue of technical complexity, massive cost, or requirement for patient's tissue. IPI is one of the traditional stratification schemes based on clinical characteristics to provide prognostic guidance for DLBCL patients. But we discovered that IPI did not represent all of the pathogenesis heterogeneity and found that an inflammatory state could also predict survival independently, and add the predictive power for IPI in DLBCL patients. A three inflammatory gene model was finally derived from this study, which was used in the stratification of the risk of death among DLBCL patients efficiently through measuring the concentration of these inflammatory‐related cytokines in peripheral blood. This model was also validated in three GEO database through detection of their mRNA expression changes in tumor tissues. The analysis procedure for this whole study was summarized in Figure S2.

A major strength of this study is the development of a predictive survival model including a small group of inflammatory genes/cytokines. During this study, the predictive power of 29 survival‐related genes, including 7 inflammatory genes, were measured based on their relative expression by RNA sequence. From the results, we identified five inflammatory genes detected in the 101 patients of DLBCL. Through the multivariate Cox regression, we designed a predictive model consisting of three genes‐ *IL6*,* IL1A*, and *CSF3*. We discovered that predictive power was correlated with the M2 TAMs infiltration, which was associated with the poor outcome of DLBCL patients.

This model divided our 101 clinical samples, 411 patients in GSE10846, 54 patients in GSE32918, and 69 patients in GSE23501 into two prognostic groups respectively. Furthermore, our method assigned the low or high IPI‐score subgroups of GSE10846 into two. This demonstrated that our method was independent of the IPI. Importantly, our predictive model could be more advantageous in individual diagnostic prognostic value than the one‐gene model through the time‐dependent ROC analysis, although each of them was reported previously to be a prognostic factor to predict the survival of DLBCL patients 7, 21, 35.

One of our important findings was that the three‐gene model could further stratify the patients with different IPI scores, thus, even among patients in the group of low IPI score, the 5 years survival rate for the high‐risk cohort identified by our three‐gene model is 58%. Moreover, in the group of high‐IPI scores, the 5‐year survival rate for the high‐risk cohort identified by our three‐gene model is 28%, suggesting a different therapeutic approach should be considered for these groups. On the basis of these data, it was worthwhile to incorporate the predictive model into the IPI score in clinical treatment.

We agree that if more genes were chosen, even with some redundancy, the predictive model may perform a better role in these independent validation analysis. However, this model is more clinically practical since it only uses a smaller number of genes.

In summary, we have described a novel independent 3‐gene model to effectively predict the prognosis of DLBCL patients by determining the concentration of these inflammatory cytokines from the peripheral blood, thus providing new insights into the association between inflammatory‐gene models and the survival of DLBCL patients.

## Conflict of Interests

The authors declare that they have no competing interests.

## Supporting information


**Figure S1.** Genes risk score analysis of entire GSE10846 series.
**Figure S2.** A flow chart showing the analysis procedure for the whole study.
**Table S1.** Univariable and multivariable Cox regression analysis in serum samples of DLBCL patients.
**Table S2.** Multivariable Cox regression analysis in our clinical cohort and GSE10846.
