# Supplementary material for: Prediction of survival of diffuse large B‐cell lymphoma patients via the expression of three inflammatory genes
Source: Cancer Med. 2016 Jul 9;5(8):1950–61. doi: 10.1002/cam4.714 (PMC4971923; doi:10.1002/cam4.714)
Supplement: Supplementary file 1 — Figure S1. Genes risk score analysis of entire GSE10846 series. Figure S2. A flow chart showing the analysis procedure for the whole study. Table S1. Univariable and multivariable Cox regression analysis in serum samples of DLBCL patients. Table S2. Multivariable Cox regression analysis in our clinical cohort and GSE10846. [file CAM4-5-1950-s001.pdf]

Prediction of survival of diffuse large B-cell lymphoma patients via the expression of three inflammatory genes

Shuangtao Zhao[1, 2], Nan Bai[3], Jianlin Cui[1,2,4], Rong Xiang[1,2,4]* and Na Li[1,2,4]*

From [1] School of Medicine, [2] Collaborative Innovation Center for Biotherapy, Nankai University, 94 Weijin Road, Tianjin 300071,China

[3]Prenatal Diagnosis Center, the First Affiliated Hospital of Zhengzhou University, Zhengzhou , Henan 450052, China

[4] Tianjin Key Laboratory of Tumor Microenvironment and Neurovascular Regulation, Tianjin 300071, China.

Corresponding author: Dr. Rong Xiang, (86)-22-23509482; Fax. (86)-22-23502554; Email: rxiang@nankai.edu.cn and Dr. Na Li, Tel. (86)-22-23509779; Fax. (86)-22-23502554; Email: lina08@nankai.edu.cn

Supplementary Information

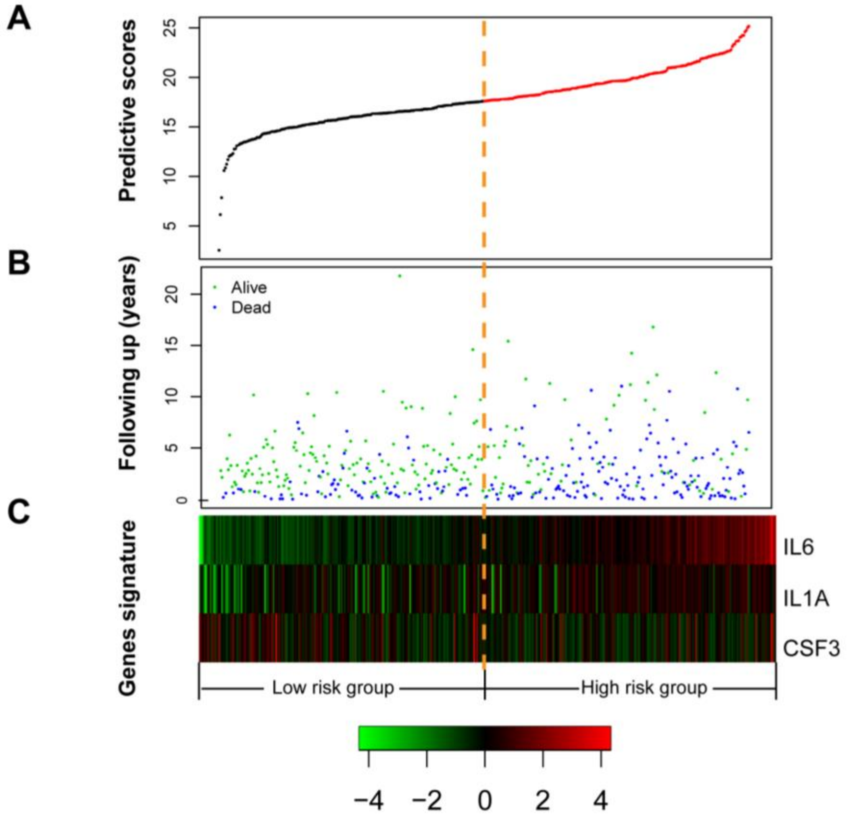

Figure S1. Genes risk score analysis of entire GSE10846 series. The distribution of three-gene predictive score, patients' survival status and genes expression signature were analyzed in the entire GSE10846 series patients (n = 411). **A.** Genes risk score distribution; **B.** Patients' survival status and time; **C.** Heatmap of the genes expression profiles, rows represent genes, and columns represent patients. The orange dotted line represents the median of three-genes risk score cutoff dividing patients into low-risk and high-risk groups.

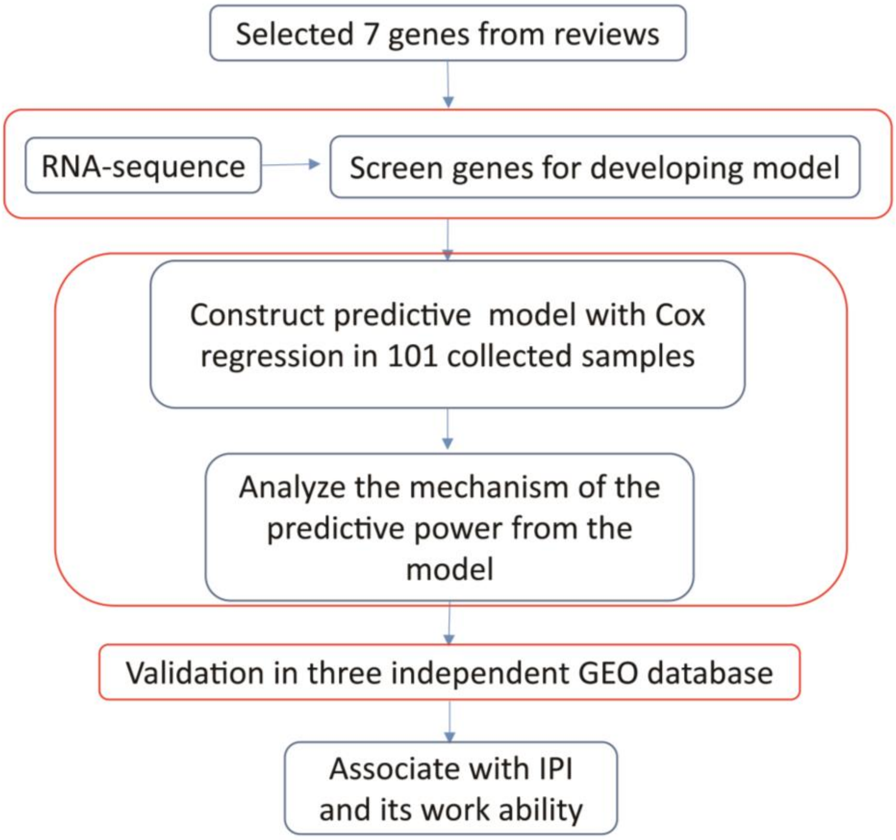

Figure S2. A flow chart showing the analysis procedure for the whole study.

Table S1. Univariable and multivariable Cox regression analysis in serum samples of DLBCL patients.

**Variables**	**Univariable model**			**Multivariable model**		
	HR	95%CI of HR	*p* value	HR	95% CI of HR	*p* value
Gender	2.243	0.181 to 27.798	0.529			
Age	1.019	0.109 to 9.559	0.987			
Ann Arbor	2.873	0.51 to 16.189	0.232			
Genotype	0.079	0.003 to 2.034	0.126	0.16	0.021 to 1.215	0.076
IL1A	2.119	0.58 to 7.742	0.256	1.98	0.946 to 4.144	0.07
IL6	13.02	1.606 to 105.529	0.016	7.299	1.709 to 31.178	0.007
IL8	0.959	0.159 to 5.777	0.964			
CSF3	0.594	0.282 to 1.254	0.172	0.467	0.228 to 0.967	0.04
CCL3	1.99	0.545 to 7.267	0.298			

Table S2. Multivariable Cox regression analysis in our clinical cohort and GSE10846.

**Group**	**Variables**	**HR**	**95%CI of HR**	***p* value**
Our cohort	Gender	37.31	0~98.71	0.965
(n=101)	Genotype	0.01	0~0.337	0.031
	IPI	11.48	0.928-142.085	0.057
	Predictive model	5.16	1.05-25.35	0.044
GSE10846	Gender	0.925	0.645-1.326	0.67
(n=411)	Genotype	1.201	0.941-1.533	0.14
	IPI	1.712	1.471-1.992	0
	Predictive model	1.073	1.008-1.141	0.026
